# A constraint-based modelling approach to metabolic dysfunction in Parkinson's disease

**DOI:** 10.1016/j.csbj.2015.08.002

**Published:** 2015-09-02

**Authors:** Longfei Mao, Averina Nicolae, Miguel A.P. Oliveira, Feng He, Siham Hachi, Ronan M.T. Fleming

**Affiliations:** a Luxembourg Centre for Systems Biomedicine (LCSB), University of Luxembourg, 7, avenue des Hauts-Fourneaux, L-4362 Esch-Belval, Luxembourg; b Department of Infection and Immunity, Luxembourg Institute of Health (LIH), 29, rue Henri Koch, L-4354 Esch-sur-Alzette, Luxembourg

**Keywords:** Dopaminergic neurons, Constraint-based modelling, Metabolic reconstruction, Energy metabolism, Parkinson's disease

## Abstract

One of the hallmarks of sporadic Parkinson's disease is degeneration of dopaminergic neurons in the pars compacta of the substantia nigra. The aetiopathogenesis of this degeneration is still not fully understood, with dysfunction of many biochemical pathways in different subsystems suggested to be involved. Recent advances in constraint-based modelling approaches hold great potential to systematically examine the relative contribution of dysfunction in disparate pathways to dopaminergic neuronal degeneration, but few studies have employed these methods in Parkinson's disease research. Therefore, this review outlines a framework for future constraint-based modelling of dopaminergic neuronal metabolism to decipher the multi-factorial mechanisms underlying the neuronal pathology of Parkinson's disease.

## 1 Introduction

After Alzheimer's disease, sporadic Parkinson's disease (PD) is the second most common neurodegenerative disorder, affecting about 0.3% of the entire population, 1% of people over 60 years of age and up to 4% of those over 80 years of age [1]. In PD, neuronal populations located within several anatomical locations appear to have different susceptibility to neurodegeneration [2,3], although the classical motor symptoms of the disease result from degeneration of dopaminergic neurons (DNs) in the substantia nigra pars compacta [1,3]. Despite intensive research, the cause and biochemical mechanisms of dopaminergic neuronal death in PD are incompletely understood. Proteostasis, oxidative stress, mitochondrial dysfunction, excitotoxicity, neuro-inflammation and more recently gut microbial dysbiosis have all been associated with PD [4–8]. However, the relationship between these processes is poorly understood, especially with regard to the causes, effects and the relative importance of each dysregulated process in PD. This review summarises some molecular pathological features of selective dopaminergic neuronal degeneration, discusses recent advances in systems-level computational approaches, and presents a framework based on several key methods of constraint-based modelling that we envisage will help unravel aetiopathogenesis of PD.

## 2 Aspects of molecular pathogenesis in Parkinson's disease

Substantia nigra DNs consume a large amount of energy to maintain a tonic electrophysiological activity in their axonal terminals within the striatum, making these cells especially vulnerable to any impairment of energy metabolism [9]. In energy metabolism, oxidisation of nutrients (e.g., glucose) is kinetically coupled to reduction of cofactors (e.g., NAD^+^ reduced to NADH, prosthetic group FAD reduced to FADH_2, NADP^+^ reduced to NADPH). In turn, oxidation of reduced cofactors is kinetically coupled to generation of energy currency metabolites (e.g., ATP, GTP). Energy currency metabolites are used to drive otherwise thermodynamically unfavourable reactions that are required for maintenance of normal cellular functions, such as scavenging of reactive oxidative species (ROS), or in the case of DNs the synthesis, release and reuptake of dopamine [10]. Oxidisation of reduced cofactors can also be used to directly to drive certain biosynthesis reactions.

Modulation of NAD^+^-dependent enzymes is currently being explored to treat neurological illnesses, e.g., the key NAD^+^-dependent enzymes SIRT1 and SIRT2, which have been associated with the α-synuclein aggregation process in PD [11]. Furthermore, in a previous study, a parenteral application of NADH in PD patients resulted in increased endogenous l-DOPA (l-3,4-dihydroxyphenylalanine) biosynthesis and alleviation of the disease motor symptoms [12].

Moreover, degenerating DNs are accompanied by an increased iron accumulation [13] and also excrete neuromelanin (NM) [14] and ROS, which are responsible for microglia activation. These factors contribute to excessive neuroinflammation, which may exacerbate neuronal death [5,15]. Recent evidence has also shown the existence of synergy between neuroinflammation in PD and gene products linked to Parkinsonian phenotypes (such as α-synuclein, parkin, Nurr1, and regulator of G-protein signalling-10) [16]. A previous study using a PD mouse model found that the activation of glial cells can induce the expression of cyclooxygenase-2 (COX-2) in DNs, enhancing the susceptibility of DNs to degeneration [17].

## 3 Computational systems approaches to dopaminergic neuronal metabolism

**Fig. 1:**
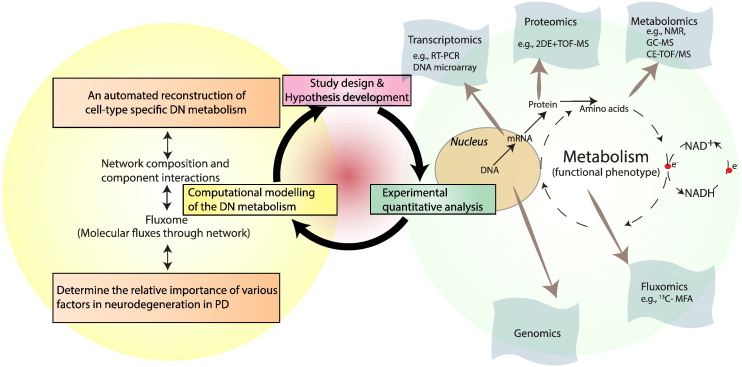
The conceptual scheme of the constraint-based modelling approach to decipher Parkinson' disease. Fluxomics quantifies the reaction rates that describe the time-dependent passage of metabolites through reactions; exometabolomics measures the abundance of primary and secondary metabolites in the extracellular environment. The modelling tasks that can be conducted by the constraint-based modelling methods are indicated by the light-yellow halo, whereas quantitative analysis that needs to be validated by experimental tools are indicated by the light-green halo. RT-PCR, reverse transcription-polymerase chain reaction; DN, dopaminergic neuron. NMR, nuclear magnetic resonance; GC–MS, gas chromatography–mass spectrometry; CE-TOFMS, capillary electrophoresis time-of-flight mass spectrometry; 2DE, two-dimensional gel electrophoresis; 13C-MFA, ^13^C metabolic flux analysis.

Elucidation of the molecular aetiopathogenesis of PD requires an interdisciplinary systems approach [18] to understand how dysfunctions of disparate pathways interact to result in neurodegeneration (Fig. 1). A systems approach consists of an iterative cycle of mathematical model formulation, computational modelling and quantitative experimental measurements. Mathematical and computational models are formal representations of biochemical knowledge that are used to propose hypotheses, design experiments and interpret experimental results. Quantitative experimental measurements are used to test hypotheses generated by a model and also generate data used to refine the content of a model.

An ultimate aim of the application of a systems approach to PD would be to be able to make non-trivial predictions of DN reactions, where quantitative modulation would either significantly imbalance normal DN energy supply, or re-balance DN energy supply in DNs generated from induced pluripotent cells derived from PD patients. At the core of this approach is an effort to reconstruct a state-of-the-art metabolic network of substantia nigra DNs, and then to apply computational modelling for generating experimentally testable hypotheses as to the aetiopathogenic nature of PD.

Network reconstruction is a prerequisite for computational modelling. A high-quality reconstruction is built from a variety of biological knowledge sources such as genome annotations, metabolic databases (e.g., KEGG and BRENDA) and biochemical literature manually curated in a quality-controlled manner [19]. These genome-scale network reconstructions provide a detailed and self-consistent representation of biochemical reaction networks and provide a basis for computation of biochemically feasible functional states using computational modelling, e.g., constraint-based modelling.

Constraint-based modelling is a scalable computational modelling approach widely used for prediction of physicochemically and biochemically feasible steady-state metabolic fluxes (reaction rates) in living cells [20]. Here, a steady state invokes the assumption that the concentrations of each metabolite are time invariant, while certain metabolites are consumed or produced by exchange reactions that translocate specific molecules across the boundary of the system being studied. This approach was successfully applied in various biomedical and biotechnological contexts, including prediction of exchange fluxes in human metabolism, e.g., [21].

Software platforms have been developed to automate certain steps of the reconstruction process [22]. However, these algorithms are mostly suited to microbial metabolic network reconstruction and need to be adapted for use when reconstructing metabolic networks in multi-cellular organisms, where the metabolic function of each cell type has evolved to be interdependent with the function of other cell types in the body. For example, in constraint-based modelling of microbial metabolism, one often optimises a biomass reaction that represents the material and energy costs associated with cell replication. In contrast, for a mature DN, which does not divide, perhaps the main metabolic objective is to maximise the rate of energy generation to meet the demand to tonically propagate action potentials over a large axonal arbor.

## 4 Constraint-based modelling of neuronal metabolism

Relatively small scale computational models were developed and applied to investigate particular aspects of DN metabolic reaction kinetics, e.g., predicting dysregulation of dopamine homeostasis [23] and the role of oxidative stress [24] in PD. Phenomenological kinetic modelling is not suited to elucidate multi-factorial mechanisms underlying neurodegeneration at genome-scale due to (i) the paucity of data on enzyme kinetic parameters, and (ii) the intractability of currently available kinetic modelling algorithms. Many of the kinetic parameters used in kinetic modelling studies are associated with large uncertainty because they were acquired from in vitro rather than in vivo measurements. Compared with kinetic modelling, constraint-based modelling approach requires fewer parameters and uses algorithms that are still tractable even when modelling large biochemical systems.

Research efforts have recently been devoted to reconstruct neuronal cell metabolism [25,26]. The latter reconstruction comprises 524 metabolites and 630 reactions, encoded by 570 genes. This network is centred to integrate general knowledge of neuronal cells and further experimental validation with metabolomics or fluxomics data. Another study reconstructed an abstract model of DN metabolism [27] consisting of only 111 metabolites and 139 reactions. However, excluding so many metabolic reactions can reduce the predictive power of the corresponding model. Furthermore, all of the aforementioned models do not take into account the fact that midbrain DNs at different anatomical locations do have distinct neurochemical phenotypes and exhibit differential vulnerability to degeneration in PD.

Comprehensive and anatomically resolved experimental gene expression datasets have recently become available, e.g., Allen brain atlas [28]. Such datasets permit new computational approaches to investigate the relationship between anatomy, cellular molecular phenotype and selective neuronal vulnerability in PD. Reconstruction of the DN metabolic network can be also greatly accelerated by the recent release of the latest genome-scale human metabolic reconstruction, i.e., Recon 2 [21]. Recon 2 is a generic reconstruction in the sense that it contains metabolic reactions from all cell types of the human body. A draft reconstruction for a particular cell type can be generated from Recon 2 by first extracting a core set of reactions that are likely to be active in that cell type, based on some set of omics data (e.g., transcriptomics, proteomics, metabolomics).

A typical set of core reactions is usually not flux consistent, i.e., not every reaction has a non-zero flux in at least one steady state flux vector. A number of methods have been developed to reconstruct context-specific models from existing genome-scale models by integrating various types of high-throughput data (as reviewed in [29]). The most recently published algorithm is FASTCORE [30], which is devised to efficiently add additional reactions to fill gaps in the cell-type-specific network in order to yield a flux consistent network. FASTCORE can eliminate the inconsistency between fluxes and gene expressions for the network reconstruction by using linear optimisation to favour fluxes through reactions with gene expression above a certain threshold and disfavour fluxes through reactions with expression lower than the threshold. The algorithm takes as input a global metabolic model and a set of reactions that is already known to be active in a given context, and it produces a context-specific model. The customised choice of flux threshold is critical as it discriminates expressed and non-expressed reactions, that is, any reactions that carry fluxes higher than the chosen threshold value are identified as expressed and must then be present in the cell-type-specific network.

### 4.1 Reconstruction of cell-type specific DN metabolism

**Fig. 2:**
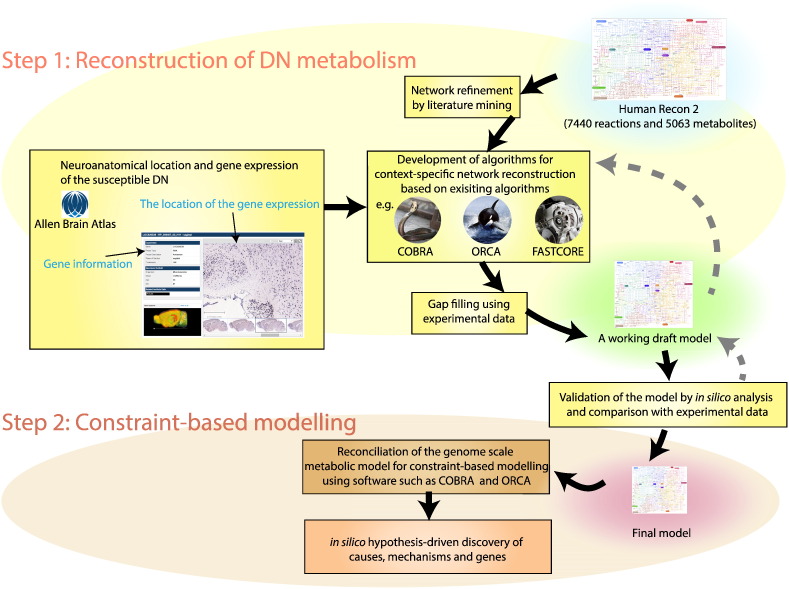
Flowchart depicting the model development steps for cell-type specific reconstruction and constraint-based analyses.

Network reconstruction is an iterative effort, and the procedure is now well established for metabolic networks [19]. A previous study developed a workflow that integrates gene expression data, proteomics data and literature-based manual curation to generate human glutamatergic, GABAergic and cholinergic mitochondrial metabolic reconstructions based on a generic human metabolic reconstruction [31]. The overall workflow for the reconstruction of DN metabolism is the same, comprising of two main stages (Fig. 2). In the stage of obtaining a draft cell-specific neuronal network, the community generated consensus reconstruction of human metabolism, i.e., Recon 2 [21], can be used as a template, then specialised and extended to represent the known metabolic functions of DNs in normal and disease states.

To accelerate the reconstruction process, the development of an automated framework is required to support the manual effort associated with literature curation. Specifically, based on existing features of established constraint-based modelling software, e.g., The COBRA toolbox [32], it is necessary to add supplementary features specific to neuronal network reconstruction. The stages of reconstruction guidelines are listed in Table 1, as well as the processes and software involved. Some steps in the reconstruction of DN metabolism can be performed by existing COBRA functions, while others require the development of new COBRA functions. The automated steps can be checked against experimental evidence, and software inputs and outputs can be manually evaluated to detect potential misspecification by automated reconstruction algorithms. Later, in the stage of validation, the reconstruction can be compared with reaction stoichiometry from previous dynamic models [24,23], non-specific neuron models [26] and an abstract DN model [27]. Previously developed software tools can be used as external simulation platforms to add notes, references and confidence scores supporting the inclusion of each reaction to the reconstruction and convert the reconstruction into the standard model exchange format (SBML Level 3).

**Fig. 3:**
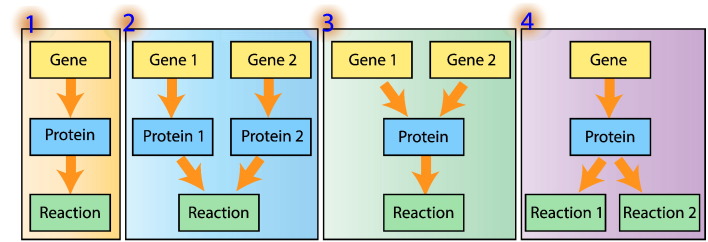
Examples of detailed gene–protein–reaction (GPR) associations. (1) Simple association, in which a single gene encodes a single enzyme. (2) Isozymes, in which multiple genes encode distinct proteins carrying out the same function. (3) Multimeric protein complex, wherein multiple genes encoding distinct protein subunits come together to form an active enzyme. (4) Multifunctional protein, in which a single protein can carry out multiple reactions.

A draft reconstruction is developed to include as large a part of cell metabolism as possible and as much biological information as possible. Several reconstruction steps, such as the genome annotation and reconstruction of metabolic pathways, can be facilitated by automated computer-based tools, using biochemical information retrieved from a number of freely or commercially available metabolic databases, such as KEGG [34], BRENDA [35], UniProt [36], and HumanCyc [37]. The determination of the gene–proteins–reaction associations (GPRs) associated with each metabolic reaction is typically conducted by a laborious and time-consuming literature search. It is thus worthwhile to develop software tools to automate the process of adding GPR rules during the reconstruction process with reference to four different ways of relating genes, proteins and reactions (Fig. 3). For example, gene annotations can be retrieved from online databases such as HumanCyc^1^. These annotations establish relationships between genes and the production of peptides. As the composition of a protein can be retrieved from databases such as UniProt,^2^ the relationship between the genes and the proteins can be determined. Then, the reactions associated to each protein can be retrieved from databases such as KEGG^3^ and BRENDA,^4^ using enzyme identifiers such as EC numbers. Therefore, GPR associations can be established by integrating inferred relationships between genes and peptides, peptides and proteins, and proteins and reactions. Parts of the integration process could be facilitated and automated by developing new software. It is also required to develop algorithms that correct the discrepancies in biological identifiers that may exist in data representations across different data sources [38]. Despite the advantages of automation, the results must be considered draft reconstruction components, until manually checked for correctness.

During a reconstruction effort, particular care must be given to the reconstruction of metabolic pathways unambiguously genetically and experimentally associated with PD, e.g., glucocerebrosidase in sphingolipid metabolism [39], and mitochondrial oxidative phosphorylation metabolism. Experimental studies of DNs in PD showed that the dysfunction of oxidative phosphorylation is due to reduced activity of NADH-ubiquinone reductase (Complex I) and NADH cytochrome C reductase [40]. Oxidative phosphorylation consumes NADH, which is supplied by three main pathways: glycolysis, TCA cycle and fatty acid oxidation, to then produce ATP, which is the fuel currency of various cellular functions. Since NADH is commonly used in reactions related to energy metabolism and was recently suggested as a reporter molecule for PD by in silico modelling [27], it is necessary to prioritise balancing these endogenous redox mediators in the context of oxidative phosphorylation, glycolysis, TCA cycle and fatty acid oxidation.

The metabolic pathways of oxidative phosphorylation can be established by defining the biochemical reactions with reference to a list of mitochondrial databases (Table 2). A reconciliation of incomplete and contradictory information from different databases is needed to obtain details about the localisation of the enzymes, the specificity of intracellular transporters and cofactor preferences. Based on data reliability, the reactions included in the cell-specific network reconstruction can be assigned different confidence scores, for example, a high score can be assigned to a reaction with strong evidence, whereas a lower score for reactions with insufficient evidence.

A table of the available ‘omics’ resources can be ranked and assembled in order of suitability, which can help leverage existing investment in the generation of data. As no published study has yet reconstructed the cell-type specific DN metabolic network at genome-scale, there is a need to establish some principles and a useful checklist to ensure the integrity and quality of the reconstruction of DN metabolism.

### 4.2 Model refinement by incorporation of experimental data

Relevant metabolomics and fluxomics data, and manual curation of biochemical literature are required to ensure the integrity and high-quality of a metabolic reconstruction (Fig. 1). With the aid of programming tools, these omics data can be translated into different types of constraints, such as, lower and upper bounds of individual reaction fluxes, reaction directions and ratios of the reaction fluxes. The incorporation of experimentally obtained datasets can improve the predictive power and reliability of the DN model by shrinking the solution space that contains all possible flux solutions calculated in constraint-based modelling.

#### 4.2.1 Fluxome

Metabolic fluxes can be estimated using ^13^C-based metabolic flux analysis (^13^C-MFA), in which intracellular fluxes are quantitatively determined by culturing cells in the presence of a ^13^C isotopically labelled carbon source and tracing the transition path of the labelled atoms between metabolites in the biochemical network using mathematical modelling [41]. In addition, the kinetic parameters of reactions can be estimated and converted into inequality bounds on metabolite concentrations in FBA modelling [42,43]. Through integration of metabolite kinetics with FBA simulations, it is possible to improve the predictive accuracy of the metabolic model by reducing the solution space of the FBA model and unravel relationships between the abundance of substrate, products, cofactors, activators and inhibitors.

#### 4.2.2 Exometabolome

Extracellular metabolite abundances (the exometabolome) can be quantified using in vitro cultures of DNs derived from human neural stem cells. By analysing the exometabolomic data, the metabolomic profile of energy metabolism can be elucidated at different metabolic states of the cell, using previously developed protocols [44]. Measured metabolite uptake and secretion rates in dopaminergic neuronal cell cultures can be used to curate a metabolic network reconstruction and fine-tune the parameters of the computational model. A recent study has developed a workflow to predict the intracellular metabolic states based on extracellular metabolomics data [45]. This is one way to improve the predictive capability of a constraint-based model for computing intracellular flux distributions.

#### 4.2.3 Manual curation of biochemical literature

Extensive manual curation of the experimental literature provides valuable data for a cell-type specific dopaminergic neuron reconstruction, especially central metabolic pathways, including glycolysis, the TCA cycle and oxidative phosphorylation. Cell-type specific gene expression data can be manually retrieved from the Allen brain atlas,^5^ which is quite time-consuming, resulting in a need to develop software and algorithms to automate certain processes, such as retrieval of neuroanatomical and neurobiochemical data for network reconstructions for other cell types such as microglia and astrocytes. In addition, exchange reaction rates could be retrieved for dopaminergic neurons from literature. These exchange reaction rates can serve as input/output constraints to a constraint-based metabolic model. They can be used for the dopaminergic neuronal metabolic reconstruction, and later for simulation of valid metabolic states for computational modelling of normal and diseased states.

#### 4.2.4 Image analysis

It is important to develop and apply software to quantify spatiotemporally resolved morphological features from fluorescent images of dopaminergic neurons in cell culture. By quantification of the abundance and morphology of dopaminergic neurons in vitro, it is possible to compare metabolomic and mass isotopomer abundance data from different cultures, by normalising to the dopaminergic neuronal cell volume in each culture. Automated incorporation of the large-scale auxiliary information is important to the PD research community to adapt or calibrate the DN model to their own case-specific simulations in the future.

Furthermore, it is necessary to qualitatively test the accuracy of a dopaminergic neuronal metabolic model, refine the reconstruction and corresponding constraint-based computational model, by comparing the pathways predicted to be active in the model with pathways established experimentally and in the literature. Many of the relevant in silico tests can be performed using existing software.

### 4.3 Determination of the relative importance of various metabolic pathways to neurodegeneration in PD

The degeneration of a single DN in PD may be the result of perturbations to energy metabolism. All else being equal, this could either be a decrease in the capacity to produce energy or an increase in demand for energy. Therefore, PD could either result from impaired function of metabolic reactions that generate energy or decreased energy efficiency in non-metabolic processes consuming energy, e.g., protein degradation. The molecular causes and metabolic pathways can be identified by understanding the relationship between metabolic reactions already hypothesised to be important in the pathogenesis of PD, e.g., mitochondrial oxidative phosphorylation, with other reactions included in the reconstruction. This can be done by calculating how a change in the rate of a given reaction would influence the rate of energy supply toward electrophysiological activity. This may uncover cascading effects of redox perturbation and over-expression of inflammation-associated enzymes such as cyclooxygenase-2 (COX-2).

The metabolic mechanisms involved in neurophysiological activities of DN can be identified by a number of methods based on sensitivity analysis [46]. These methods can be implemented to analyse the trade-off between different metabolic functions of the DN and identify the maximum reaction rates of the phenotypical behaviours allowed by physical constraints such as mass balance, with respect to perturbation in the rates of each metabolic reaction.

Specifically, robustness analysis, as exemplified by previous studies [47,48], can be performed to test how DN metabolism adjusts its energy regeneration mechanisms to meet the known high demand for energy consumption in a DN [49], while maintaining a steady state. The network robustness is related to the topology of the metabolic model and can be described by the functional and dynamic changes that result from perturbations. Robustness analysis can give an insight into the relationship between available energy sources and enzyme fluxes at an objective-oriented metabolic state.

The steady states can be further constrained by metabolic uptake rates and the net direction of each metabolic reaction. Further thermodynamic constraints can be added such that steady states are also thermodynamically feasible. Energy balance analysis [50-52], a complementary method to FBA, can constrain the solution space and improve the accuracy of the FBA theoretical prediction by taking into account thermodynamic principles. However, it is noteworthy that energy balance analysis can only be conducted on small or medium-sized networks and requires the biochemical thermodynamic parameters of all reactions. Out of this set of steady states, strictly convex optimisation algorithms [53] can be used to predict a unique steady state reaction rate for each metabolic reaction, where it is possible to set multiple weighted optimisation objectives [54], based on known metabolic demands, e.g., energy demand for electrophysiological activity.

There is no human biomass equation available in the literature and a neuron does not proliferate like a cancer cell or a microorganism. The objective of the neural cell is thought to achieve different metabolic functions, such as secretion of a range of metabolites into surrounding environments. Different objectives have been used in studies applying constraint-based modelling to calculate an optimal flux distribution. These objectives include maximisation of fluxes through apoptosis, protein degradation, ATP, O2, α-synuclein and tyrosine production pathways [27], maximisation of the sum of the fluxes through glutamate/glutamine/GABA cycles [26], and maximisation of the energy metabolism to increase dopamine release required for neuronal signalling [55].

In contrast to single-objective based studies, it is preferable to employ Pareto optimality analysis [56] to reveal the competitive and synergic effects among these metabolic functions. This method allows the evaluation of the impact of any enhanced metabolic activities on the cellular metabolism. Optimisation of a number of competing objectives is a well-studied problem and gives rise to the concept of a Pareto front [56], which is a range of solutions that interpolate between a number of extremes. At each extreme, one objective is optimised on its own; in between, the objectives are combined in varying proportions. The objective equation can be formulated as described in previous studies [33,47,48]. Briefly, a multi-objective equation comprises: 1) one objective that represents the maintenance energy cost and 2) other objectives that reflect different metabolic functions of the neuron. Use of a multi-objective with adjustable relative contributions is a standard computational device and was also evaluated for metabolic modelling [57]. Practical calculations involve maximising a weighted combination of objective fluxes for a series of fixed λ values — the weighting factor for the importance of the objective. As commonly found, equally spaced λ values do not yield equally spaced points on the Pareto front. Suitable values can therefore be determined by construction of an equispaced Pareto front using a sequence of optimisation problems as exemplified in a previous study [56]. In the present context, λ = 0 represents the metabolic state where flux through the associated objective is not encouraged by the optimisation, while λ = 1 is the other (in practice non-viable) extreme where all metabolic resources are devoted to achieving the objective. Equal weighting may not necessarily represent actual physiological priorities, but it is appropriate to evaluate how the objectives mutually influence each other. In this sense, the value of λ can also be interpreted as a measure of the degree of perturbation caused by different objectives — metabolic functions of the DNs.

Then, a fractional benefit analysis, as described previously [47,48], can be conducted to quantify additional benefit of the trade-off to metabolism between the multiple objectives — how each of the multiple objective terms contributes (positively or negatively) to the overall benefit. It can also be used to quantify the competitive or synergic effect between different objectives, for example, ATP and dopamine production.

Furthermore, a number of PD-associated genes have already been reported, such as those encoding α-synuclein, parkin, PINK1, DJ-1, LRRK2, and ATP13A [58]. Using the available biochemical knowledge, it is therefore possible to perform sensitivity analysis for specific reactions in the metabolic reconstruction to unravel relationships between the expression of genes encoding these reactions, which are functionally related to the PD-associated genes, and the normal metabolic functions under the pathogenesis of PD diseases.

## 5 Summary and outlook

PD has a multi-factorial aetiology, involving interaction between multiple perturbed biochemical reactions. The latest human generic metabolic reconstruction [21] can be combined with existing and newly generated high-throughput experimental data to develop a comprehensive reconstruction of the dopaminergic neuronal metabolism. Development of a constraint-based computational model, derived from the aforementioned reconstruction, is envisaged to predict the set of metabolic pathways critical for maintenance of normal dopaminergic neuronal metabolic function and predict the combination of genetic perturbations that confer selective vulnerability to degeneration of DNs.

There is a shortage of research activities in the DN reconstruction and modelling effort. The reconstruction requires not only the manual reconstruction from the classical neurobiochemical literature, but also a parallel and complementary effort to apply and adapt existing tools for automated reconstruction of metabolic networks in order to accelerate the comprehensiveness of the existing DN reconstruction and modelling effort. This permits the incorporation of high-throughput experimental data, which can be used as an independent means to establish the activity of individual reactions within DN susceptible to degeneration in PD. Once the parallel manual and automated reconstruction effort has resulted in an integrated computational model, it is possible to combine biochemistry and computational modelling to determine the relative importance of various factors implicated in neurodegeneration in PD.

There are several potential challenges in the DN reconstruction. First, the pipeline methods for network reconstruction are well described by previous studies [19] but many of these methods were developed for microbial metabolism reconstruction. It is thus necessary to identify the difference between microbial and neuronal reconstructions, and to develop a new computational framework based on existing published methods, which could involve some unforeseen problems pertaining to the metabolic difference between DNs and other cell types. Second, one necessary step during metabolic reconstruction is establishing a good representation of cellular objectives of the DN for FBA modelling. This could involve many iterations of in silico testing and verification, as well as comparison with literature and experimental data. In addition, the quality of the objective function could be undermined by the experimental error and small sample size of measured data. Third, the metabolic reconstruction relying on multiple biological databases and different sources of experimental data may involve inconsistencies or errors (such as in metabolite naming), which could require extensive efforts to reconcile and develop methods to standardise the generalised databases for the DN reconstruction. Therefore, to ensure the integrity and quality of the reconstruction of DN metabolism, it demands the combined efforts among computational biologists and biochemists.

A range of age-related processes (e.g., insulin resistance, diabetes and obesity) might be linked to the pathophysiological mechanisms of PD, via natural and chronic alterations in whole-body metabolism [59]. Incorporation of age-related alterations into the computational model may help elucidate the interplay between age- and PD-related processes. To unravel the complex relationships between neurodegeneration and peripheral alterations in PD, it may also be worthwhile to develop interaction models based on metabolic reconstructions of DN and other cells in non-dopaminergic associated areas. These models may be used to study the interaction between dopamine pathways in neurons and inflammatory and cell death pathways in various types of glial cells [60] in order to provide a more complete picture of the aetiology and pathogenesis of PD.

It may be worth noting that the prevalence of PD is 1.5–2 times higher in men than women [61-64]. Therefore, a future direction for constraint-based modelling of DNs in PD is to develop gender-specific metabolic models to identify underlying mechanisms linking to the differences between the two sexes. These metabolic models can be reconstructed based on the omics data from literature mining for sex differences in metabolism [65-67] and gene expression profiling of male and female human induced pluripotent stem cells (iPSCs) [44,68], and has potential to facilitate the design of gender-related therapies for PD patients.

## Competing interests

The authors declare that they have no competing interests.

## Figures and Tables

**Table 1:** The stages of the published reconstruction guidelines and implementation of the software platform.

Stages of the reconstruction guideline	Required activities for constraint-based modelling of the DN metabolism	Available software tools or actions
Draft reconstruction	Assemble a draft reconstruction using Recon2 as template and include candidate metabolic reactions and functions.	Context-specific metabolic network reconstruction algorithms [29] e.g., FASTCORE
Refinement/curation	Determine metabolic functional requirements (e.g., dopamine production, maintaining tonic firing at different frequencies)	No suitable software
Add transport reactions/constraints to represent the transit between compartments	
Refinement and assignment of GPR rules	COBRA functions
Reconstruction of metabolic pathways for neuronal mitochondrial phosphorylation. Balancing different pairs of redox cofactors, including: NADH/NAD^+^, NADPH/NADP^+^ and ATP/ADP balancing.	Require to develop new functions based on available software
Database and information integration, for example, retrieval of metabolites and biochemical reactions from a range of mitochondrial databases	
Determine substrate usage and cofactors	
Determine and add confidence scores	
Add references and notes	
Flag cell-type-specific information on DN	
Add ATP-maintenance reaction	
Conversion to a genome-scale model		COBRA functions
Network evaluation	Test for stoichiometrically balanced cycles (no software can yet test for such cycles universally)	Identifiable by ORCA [33] and resolved by COBRA functions
Test the production of different precursors	Refinement of existing COBRA functions
Test different physiological properties	Refinement of existing COBRA functions
Relationships between competing functions	ORCA functions
Model-driven discovery	Multi-objective based sensitivity analysis to identify reactions supporting the neurophysiological activities	ORCA functions
Test the robustness of the metabolic reconstruction	COBRA functions
Identify the genes linked to PD aetiology	COBRA functions

**Table 2:** List of useful databases for reconstruction of DN mitochondrial sub-network and data analysis.

Database	Function	Website
*Genome annotation*		
Mitocarta	An inventory of 1098 mouse genes encoding proteins with strong support of mitochondrial localisation.	http://www.broadinstitute.org/pubs/mitocarta/

*Proteins/nucleotides*		
Mitop2	Search for comprehensive information of mitochondrial proteins in human	http://www.mitop2.de/
Mitominer	An integrated web resource of mitochondrial proteomics	http://mitominer.mrc-mbu.cam.ac.uk
Mitoproteome	A collection of human mitochondrial protein sequences generated from information obtained from a comprehensive curation of public databases as well as from direct experimental evidence.	http://www.mitoproteome.org/
Hmpdb	Contains comprehensive data on mitochondrial and human nuclear-encoded proteins involved in mitochondrial biogenesis and function.	http://bioinfo.nist.gov/

*Functional/pathway*		
Mitophenome	Search for genes and genetic variation and their effects on clinical disease phenotypes.	http://www.mitoproteome.org/

## Footnotes

1 http://humancyc.org.

2 http://www.uniprot.org.

3 http://www.genome.jp/kegg/.

4 http://www.brenda-enzymes.org.

5 http://www.brain-map.org.
